# Assessment of preparedness and proficiency in basic and advanced life support among nursing professionals: a cross-sectional study

**DOI:** 10.3389/fmed.2024.1328573

**Published:** 2024-01-22

**Authors:** Juan S. Izquierdo-Condoy, Fabián D. Arias Rodríguez, Erick Duque-Sánchez, Nicolás Alegría N., Marlon Rojas Cadena, Patricio Naranjo-Lara, Alexander Puglla Mendoza, Jackson Jima-Sanmartín, Dayana Andrade Casanova, Balbina García, Natalia Castaño Giraldo

**Affiliations:** ^1^One Health Research Group, Faculty of Medicine, Universidad de las Américas, Quito, Ecuador; ^2^Área de Gestión de Docencia e Investigación, Hospital Pediátrico Baca Ortiz, Quito, Ecuador; ^3^Facultad de Medicina, Corporación Universitaria Empresarial Alexander von Humbolt, Armenia, Colombia

**Keywords:** cardiopulmonary resuscitation, basic life support, advanced life support, nursing professionals, academic training

## Abstract

**Background:**

Cardiac diseases are among the leading causes of death worldwide, including sudden cardiac arrest in particular. Nursing professionals are often the first to encounter these scenarios in various settings. Adequate preparation and competent knowledge among nurses significantly impact survival rates positively.

**Aim:**

To describe the state of knowledge about Basic and Advanced Life Support guidelines among Ecuadorian nursing professionals.

**Methodology:**

A nationwide, descriptive, cross-sectional study was conducted from February to April 2023 among Ecuadorian nursing professionals. Participants were invited through official social media groups such as WhatsApp and Facebook. The study utilized a self-administered online questionnaire to evaluate theoretical knowledge of Basic Life Support (BLS) and Advanced Life Support (ALS). Knowledge scores were assigned based on the number of correct answers on the tests. T-tests and one-way ANOVA were used to examine relationships between knowledge scores and demographic and academic training variables.

**Results:**

A total of 217 nursing professionals participated in the study. The majority of the participants were female (77.4%) and held a university degree (79.9%). Among them, only 44.7% claimed to have obtained a BLS training certificate at least once, and 19.4% had ALS certification. The overall BLS knowledge score (4.8/10 ± 1.8 points) was higher than the ALS score (4.3/10 ± 1.8 points). Participants who had obtained BLS certification and those who used evidence-based summaries as a source of extracurricular training achieved higher BLS and ALS knowledge scores.

**Conclusion:**

Ecuadorian nursing professionals in this study exhibited a significant deficiency in theoretical knowledge of BLS and ALS. Formal training and preparation positively impact life support knowledge. Support and inclusion of Ecuadorian nurses in training and academic preparation programs beginning at the undergraduate level are essential for promoting life support knowledge and improving outcomes.

## 1 Introduction

Heart disease is a leading cause of death worldwide, and sudden cardiac arrest is a critical emergency affecting individuals across all age groups (1). Timely, life-saving interventions are essential for preventing deaths related to sudden cardiac arrest. Globally, approximately 92% of individuals experiencing out-of-hospital cardiac arrest do not survive, often due to limited access to cardiopulmonary resuscitation (CPR) (2). Out-of-hospital cardiac arrest significantly contributes to both mortality and disability, accounting for up to 10% of total mortality in developing countries (3, 4). Temporal trends in recent years have shown that more and more bystanders are practicing CPR, however this trend has not necessarily been reflected in the survival outcomes of those affected, highlighting the importance of optimal preparation and correct practice of CPR in these scenarios (5).

Basic Life Support (BLS) entails recognizing various conditions such as myocardial infarction, sudden cardiac arrest, foreign-body airway obstruction, and administering CPR and defibrillation using an automated external defibrillator (4). The American Heart Association (AHA) emphasizes that prompt CPR and defibrillation within the first 3–5 min of collapse can substantially increase survival rates, ranging from 49% to 75% of cases (6, 7). Successful BLS implementation can double or even triple survival rates for witnessed sudden cardiac arrest (8). Therefore, healthcare workers’ knowledge and awareness of BLS are of utmost importance (9–11).

On the other hand, Advanced Life Support (ALS) involves not only high-quality CPR but also a series of actions aimed at restoring spontaneous circulation as quickly as possible. These actions include invasive interventions, such as endotracheal intubation and the use of intravenous catheters for fluid and drug administration (12). ALS focuses on treating underlying causes and providing support during post-cardiac arrest recovery. Optimizing the chain of events that must occur in rapid succession to maximize the chances of survival from sudden cardiac arrest (chain of survival) is crucial for ensuring functional survival for adults experiencing cardiac arrest (10).

Nursing professionals regularly encounter emergency cases, including sudden cardiac arrest, in both hospital and non-hospital settings (10, 13). In these scenarios, among healthcare personnel, nurses have been identified as key first responders capable of performing BLS. Studies have shown that nurses effectively applying first aid and BLS techniques in acute conditions positively impact morbidity and mortality rates related to cardiac arrest (13). However, it has been observed that both knowledge and skills tend to fade over time, with ALS skills deteriorating more rapidly than BLS skills. This underscores the ongoing need for regular training and resource utilization to reinforce these skills (14, 15).

In this context, various studies have highlighted deficiencies in life support skills among nursing professionals. For example, a study in India assessed skills and knowledge in BLS and ALS among nursing staff, revealing an average score of 9 out of 20 points (16). Additionally, De Lima et al. found that only 65% of nursing professionals possess knowledge of the sequential actions required in BLS and ALS, and their understanding of medication preparation and administration was particularly low (17).

In Ecuador, nurses and doctors are expected to manage cardiac arrest according to standards accepted by providers, despite the limited availability of cardiac arrest equipment in most public hospitals (18). However, to date, the knowledge of nursing professionals regarding life support in Ecuador has not been explored. Thus, the objective of this study is to evaluate the level of knowledge about both basic and advanced life support resuscitation guidelines among nurses in Ecuador.

## 2 Materials and methods

### 2.1 Study design

A descriptive, cross-sectional, nationwide study was performed using an online questionnaire.

### 2.2 Setting and participants

The population for this research consisted of nursing graduates in Ecuador. To obtain a bachelor’s degree in nursing, applicants need to successfully complete nine semesters (equivalent to four and a half years) of university studies (19).

For this research, an anonymous online survey was administered to licensed nurses residing in Ecuador from February 2023 to April 2023. Study participants were selected using a non-probability convenience sampling method.

### 2.3 Data measurement and questionnaire

The research team formulated a structured questionnaire to assess the knowledge levels of Ecuadorian nurses in BLS and ALS. The questions were developed based on the theoretical foundations of BLS and ALS, as outlined in the questionnaire used by Passali et al. (13) and the 2020 American Heart Association (AHA) guidelines for BLS and ALS (10), The questionnaire also captured demographic and work-related data of the participants.

Prior to the main study, a pilot study was conducted among 15 Ecuadorian nurses to identify potential comprehension issues or structural errors in the questionnaire through feedback from the respondents. Following the revision of some questions based on the pilot study’s findings, a 35-item questionnaire was finalized in Spanish and subsequently validated by three experts in emergency medicine. An English version was also prepared for inclusion in this report (Supplementary material).

The finalized online questionnaire comprised four sections:

- The first section included 7 questions concerning demographic variables such as gender, age, academic level, and workplace.
- The second section consisted of 8 questions that evaluated participants’ training and practice in BLS and ALS, including: (1) life support training during college; (2) previous BLS certification; (3) previous ALS certification; (4) BLS practice; (5) ALS practice; (6) extracurricular life support preparation.
- The third section featured 10 multiple-choice questions to assess the theoretical knowledge of BLS.
- The fourth section had 10 multiple-choice questions to measure the theoretical knowledge of ALS.

Data were collected via Google Forms, a freely accessible web tool. Participants accessed the questionnaire through a unique link disseminated by the research team via social media platforms like Facebook and WhatsApp. The initial section of each questionnaire provided a brief overview of the study’s purpose and confirmed the confidential treatment of collected data. Informed consent was obtained from all participants, and the collected data remained anonymous.

### 2.4 Bias

To address potential biases, precautions were taken at various stages of data collection and analysis. Duplicate response bias was minimized by limiting each IP device to a single response using Google Forms’ built-in features. Furthermore, independent analysis by multiple researchers was conducted to minimize analytical bias.

### 2.5 Study size

The required sample size was calculated using a specific equation for finite or known populations (20):


$$n=\frac{\left(N.Z^{2}\right).\left(p.q\right)}{d^{2}.\left(N-1\right)+Z^{2}.p.q}$$

Based on Ecuador’s total nurse population in 2019 (*N* = 25,900) (21), and assuming a 95% confidence level, a 7% margin of error, and a 50–50 positive–negative response distribution, a sample size of 195 participants was determined.

### 2.6 Data management

Within the demographic variables, the work sector was categorized as either public or private, based on the source of funding for the workplace. Single-choice responses were employed to evaluate participants’ training and their perceptions of knowledge in BLS and ALS.

The participants’ level of knowledge in both BLS and ALS was gauged using a decimal-type numerical rating scale, ranging from 0 to 10 points for each questionnaire (22), A value of 1 point was assigned for each correctly answered question, while incorrect answers were assigned a value of 0 points; incorrect answers did not result in point deductions. Consequently, the maximum score a participant could achieve on either the BLS or ALS questionnaire was 10 points, and the minimum score was 0 points.

### 2.7 Statistical analysis

Categorical variables were analyzed using frequencies and percentages, while quantitative variables were assessed through measures of central tendency (mean) and dispersion (standard deviation).

To search for relationships of association between the qualitative variables “sex,” “work sector,” “training during university,” “BLS certification,” “ALS certification,” “believe of sufficient knowledge of BLS” and “believe of sufficient knowledge of ALS,” with the knowledge BLS and ALS scores the Student’s T-test was used. While, searching for association relationships between multicategory variables such as “age,” “academic level,” “place of work,” “hospital work area,” “work experience,” “BLS practice,” “ALS practice” and “extracurricular training” with BLS and ALS knowledge level score, a one-way ANOVA analysis was performed, using the variables “BLS score” and “ALS score” as dependent variables. In all cases analyses with *p*-value < 0.05 were accepted as statistically significant. Results analysis was carried out in the IBM SPSS version 26.0 software.

### 2.8 Ethics approval and consent to participate

All collected information was anonymized, and participation was voluntary. Ethical approval was obtained from the Ethics Committee of the Hospital San Francisco de Quito, under the code CEISH-HGSF-2023-011, ensuring compliance with ethical guidelines and standards.

## 3 Results

### 3.1 Demographic and work characteristics

A total of 217 licensed nurses participated in this study. Of these, 77.4% (*n* = 168) were women, and 59.9% (*n* = 130) fell within the age range of 20–30 years. Concerning academic qualifications, the majority held a university degree (79.9%). Regarding professional employment, the majority worked in public sector health centers (72.8%), primarily in the first (41.0%) and second (38.7%) levels of care (Table 1). The main areas of work among respondents were outpatient consultations (32.3%) and clinical specialty areas (22.6%).

**Table 1:** Demographic characteristics of Ecuadorian nurses.

		*n*	(%)
Sex	Male	49	22.6
	Female	168	77.4
Age (years)	20–30	130	59.9
	31–40	46	21.2
	41–50	21	9.7
	50–65	20	9.2
Academic level	University degree	173	79.7
	Specialist	17	7.8
	Master’s degree	27	12.4
Level of care	First level	89	41.0
	Second level	84	38.7
	Third level	44	20.3
Work sector	Public	158	72.8
	Private	59	27.2
Hospital work area	Administrative	9	4.1
	Outpatient consultation	70	32.3
	Clinical specialty areas	49	22.6
	Surgical specialty areas	33	15.2
	Emergency	47	21.7
	Intensive care unit	9	4.1
Work experience	Less than 3 years	128	59.0
	3 to 6 years	27	12.4
	6 to 10 years	23	10.6
	More than 10 years	39	18.0

### 3.2 Life support training and preparation

In terms of training in basic and advanced life support, 82.5% of participants indicated that they had received some form of life support training during their university education. Although 16.6% of respondents had not utilized any extracurricular resources for their life support training, 30.4% reported watching videos for this purpose (Table 2). However, when it comes to BLS, only 44.7% claimed to have received a BLS certification at least once (Table 2).

**Table 2:** Preparation and training in BLS and ALS among Ecuadorian nurses.

		*n*	(%)
Training during university	No	38	17.5
	Yes	179	82.5
BLS certification	No	120	55.3
	Yes	97	44.7
BLS practice	Less than 3 times	93	75.6
	Between 3 to 6 times	19	15.5
	More than 6 times	11	8.9
Believe you have sufficient knowledge of BLS	No	101	46.5
	Yes	116	53.5
ALS certification	No	175	80.6
	Yes	42	19.4
ALS practice	Less than 3 times	59	86.8
	Between 3 to 6 times	7	10.3
	More than 6 times	2	2.9
Believe you have sufficient knowledge of ALS	No	124	57.1
	Yes	93	42.9
Extra-curricular training	Scientific articles	36	16.6
	Official Guides	46	21.2
	Evidence-based summaries	2	0.9
	Video	66	30.4
	Other	31	14.3
	None	36	16.6

For advanced life support, 80.6% of nurses had not obtained ALS certification, and over half (57.1%) did not consider themselves adequately knowledgeable in this area (Table 2).

### 3.3 Basic life support knowledge

The mean theoretical knowledge score for BLS among participants was 4.8/10 ± 1.8 points. This was higher than their mean score for ALS, which was 4.3/10 ± 1.8 points (*p* = 0.001).

Upon examining the influence of various participant characteristics, we found that neither demographic nor job-related factors significantly impacted knowledge scores (Table 3). Nevertheless, training during university education and acquiring a BLS certification were associated with a significant improvement in scores, with mean increases of 0.7/10 and 0.6/10 points, respectively (Table 3). Intriguingly, practicing real-life BLS was correlated with lower knowledge scores: for those who practiced fewer than three times, the score was 5.3/10 ± 1.7 points; for three to six times, it was 4.7/10 ± 1.5 points; and for more than six times, it was 4/10 ± 1.5 points (*p* = 0.040) (Table 3).

**Table 3:** Mean difference of BLS knowledge score and demographic and training characteristics of the participants.

Variable		BLS knowledge score over 10 points		
		Mean	± SD	*p* value
Sex	Male	4.7	± 2.0	0.524
	Female	4.9	± 1.8	
Age (years)	20–30	5.0	± 1.9	0.365*
	31–40	4.8	± 1.6	
	41–50	4.5	± 1.6	
	50–65	4.4	± 1.7	
Academic level	University degree	4.9	± 1.7	0.424*
	Specialist	4.3	± 2.2	
	Master’s degree	4.8	± 1.9	
Level of care	First level	4.9	± 1.8	0.859*
	Second level	4.9	± 1.8	
	Third level	4.7	± 1.8	
Work sector	Public	4.7	± 1.8	0.099
	Private	5.2	± 1.9	
Hospital work area	Administrative	5.0	± 2.5	0.409*
	Outpatient consultation	4.8	± 1.7	
	Clinical specialty areas	4.7	± 2.0	
	Surgical specialty areas	5.4	± 1.8	
	Emergency	4.8	± 1.6	
	Intensive care unit	4.0	± 1.7	
Work experience	Less than 3 years	4.8	± 1.9	0.225*
	3–6 years	5.4	± 1.7	
	6–10 years	4.7	± 1.3	
	More than 10 years	4.5	± 1.7	
Training during university	No	4.3	± 1.8	**0.039**
	Yes	5.0	± 1.8	
BLS certification	No	4.6	± 1.8	**0.020**
	Yes	5.2	± 1.8	
BLS practice	Less than 3 times	5.3	± 1.7	0.040*
	Between 3 to 6 times	4.7	± 1.5	
	More than 6 times	4.0	± 1.5	
Believe you have sufficient knowledge of BLS	No	4.7	± 1.9	0.159
	Yes	5.0	± 1.7	
ALS certification	No	4.8	± 1.8	0.520
	Yes	5.0	± 1.9	
ALS practice	Less than 3 times	5.1	± 1.9	0.176*
	Between 3 to 6 times	4.0	± 1.8	
	More than 6 times	6.5	± 1.7	
Believe you have sufficient knowledge of ALS	No	4.8	± 1.9	0.880
	Yes	4.9	± 1.7	
Extra-curricular training	Scientific articles	4.9	± 2.1	0.001*
	Official guides	5.5	± 2.0	
	None	3.8	± 1.6	
	Other	4.8	± 1.4	
	Evidence-based summaries	7.0	± 1.4	
	Video	4.9	± 1.6	

* *p* values calculated from one-way ANOVA test.Bold are used for *p* values < 0.05.

### 3.4 Advanced life support knowledge

Regarding theoretical knowledge in ALS, participants aged between 41 and 50 years had a higher mean knowledge score of 5.0/10 ± 1.9 points (*p* = 0.038). Similarly, those with 6–10 years of work experience scored higher, with a mean of 4.8/10 ± 1.4 points (*p* = 0.044) (Table 4).

**Table 4:** Mean difference of ALS knowledge score and demographic and training characteristics of participants.

Variable		ALS knowledge score over 10 points			Mean	± SD	*p* value
Sex	Male	4.0	± 2.0	0.214
	Female	4.4	± 1.7	
Age (years)	20–30	4.1	± 1.9	0.038*
	31–40	4.4	± 1.6	
	41–50	5.0	± 1.9	
	50–65	4.6	± 1.3	
Academic level	University degree	4.2	± 1.7	0.252*
	Specialist	4.6	± 1.7	
	Master’s degree	4.7	± 2.2	
Level of care	First level	4.3	± 1.9	0.940*
	Second level	4.3	± 1.7	
	Third level	4.2	± 1.9	
Work sector	Public	4.2	± 1.7	0.425
	Private	4.4	± 2.1	
Hospital work area	Administrative	4.1	± 2.5	0.976*
	Outpatient consultation	4.3	± 1.9	
	Clinical specialty areas	4.2	± 1.8	
	Surgical specialty areas	4.5	± 1.6	
	Emergency	4.2	± 1.8	
	Intensive care unit	4.4	± 1.4	
Work experience	Less than 3 years	4.0	± 1.9	0.044*
	3–6 years	4.5	± 1.9	
	6–10 years	4.8	± 1.4	
	More than 10 years	4.7	± 1.6	
Training during university	No	4.1	± 1.7	0.573
	Yes	4.3	± 1.8	
BLS certification	No	4.0	± 1.8	**0.003**
	Yes	4.7	± 1.8	
BLS practice	Less than 3 times	4.5	± 1.8	0.417*
	Between 3 to 6 times	4.5	± 1.7	
	More than 6 times	5.3	± 2.1	
Believe you have sufficient knowledge of BLS	No	4.2	± 1.8	0.311
	Yes	4.4	± 1.8	
ALS certification	No	4.2	± 1.8	0.100
	Yes	4.7	± 2.0	
ALS practice	Less than 3 times	4.7	± 2.1	0.984*
	Between 3 to 6 times	4.7	± 1.5	
	More than 6 times	5.0	± 2.8	
Believe you have sufficient knowledge of ALS	No	4.3	± 1.7	0.769
	Yes	4.3	± 1.9	
Extra-curricular training	Scientific articles	3.7	± 1.8	<0.001*
	Official guides	4.8	± 1.8	
	None	3.4	± 1.7	
	Other	5.1	± 1.3	
	Evidence-based summaries	6.5	± 2.1	
	Video	4.3	± 1.8	

* *p* values calculated from one-way ANOVA test.Bold are used for *p* values < 0.05.

In terms of preparation, interestingly, participants certified in BLS scored higher, averaging 4.7/10 ± 1.8 points (*p* = 0.003), compared to those who were not certified 4.0 ± 1.8 points. For ALS certification, although individuals who claimed to be certified in ALS had higher scores than those who were not, the difference was not statistically significant (Table 4). Lastly, nurses who used evidence-based summaries for extracurricular training achieved the highest score, with an average of 6.5/10 ± 2.1 points (*p* < 0.001) (Table 4).

## 4 Discussion

Given the significant impact that cardiopulmonary arrest and sudden death have on global healthcare systems, healthcare professionals—particularly physicians and nurses—play a pivotal role in mitigating the effects of these emergencies on affected individuals (23, 24). Therefore, medical and nursing professionals need a comprehensive understanding of cardiopulmonary resuscitation to execute timely and effective interventions (25, 26). To the best of our knowledge, this is the first study evaluating the theoretical understanding of life support among Ecuadorian nurses. Our findings indicate that most participants are young, have limited work experience, and have not pursued further academic specialization beyond obtaining a bachelor’s degree in nursing. Additionally, the predominant gender among participants was female (77.4%), aligning with trends observed worldwide among nursing professionals (27–31).

Despite the clear importance of life support training, as of 2023, Basic Life Support (BLS) and Advanced Life Support (ALS) training are not mandatory components of nursing curricula in Ecuadorian universities (32). This absence is evident, with 17.5% of participants reporting not having received any life support training during their university studies. Furthermore, less than half of the participants reported holding official certifications in BLS (44.7%) and ALS (19.4%). Paradoxically, 53.3% and 42.9% of participants self-perceived having sufficient levels of knowledge in BLS and ALS, respectively. This demonstrates a higher level of confidence in their knowledge despite lacking official certifications, which may underscore an underestimation of the importance of formal life support training for nursing professionals in Ecuador. This underestimation could originate from the beginning of their university training since life support is not part of their academic curricula, as mentioned.

In terms of actual knowledge levels, Ecuadorian nurses scored suboptimally on both BLS and ALS tests, averaging 4.8/10 ± 1.8 and 4.3/10 ± 1.8 points, respectively. These scores align with similarly low BLS, and both BLS and ALS knowledge levels reported in studies involving nurses from South Africa, Yemen, and Uganda, who scored 46/100, 53.65/100, and 53.8/100, respectively (31, 33, 34).

Demographic factors like age and gender, as well as job characteristics such as work area and experience, did not significantly influence BLS knowledge scores. This finding is congruent with studies conducted on South African and Egyptian nurses (30, 31). However, participants who had undergone various forms of BLS training, including undergraduate education and certification, exhibited improved knowledge levels in BLS (*p* < 0.05).

Concerning ALS knowledge, although the mean score was still lower than that for BLS, older participants (aged 41 to 50) exhibited a higher level of knowledge in ALS. A similar positive effect was observed among nurses with over 6 years of work experience. Certification in both BLS (*p* = 0.003) and ALS (*p* = 0.100) also corresponded with higher scores. We believe that the discrepancy found regarding the significance of these differences in knowledge in BLS and ALS certification contexts could be due to the lack of consideration for the time that has elapsed from certification to the time of examination. In several cases, participants with ALS certification may have obtained it either a considerable time ago or more recently. In this context, the high level of knowledge observed in those who have been certified can be attributed to various intervening variables, including the experience gained through daily professional practice, often referred to as the ‘hidden curriculum,’ since the time of certification.

An intriguing aspect of this research is the high level of self-perceived sufficiency in knowledge of BLS and ALS among the participants, even though the average knowledge scores in both areas were deficient. Specifically, BLS scores averaged 5.0/10 ± 1.7 points, and ALS scores averaged 4.4/10 ± 1.8 points. This discrepancy may suggest a level of overconfidence in the participants’ abilities, a phenomenon also noted in another study involving healthcare professionals, including nurses (35). Further analysis is required to substantiate this observation.

It has been established that the level of knowledge concerning CPR guidelines among nursing professionals is influenced by motivation, attitude, and willingness to learn (31). This is corroborated by our study, which revealed that nurses using evidence-based summaries for extracurricular training achieved significantly higher scores in both BLS (*p* = 0.001) and ALS (*p* < 0.001).

Despite the global emphasis on the critical role that nurses play in life support settings, our study indicates deficiencies and inadequacies in training initiatives in Ecuador, both during undergraduate education and through official programs. It is evident that these shortcomings result in deficient levels of knowledge, a concern heightened by the fact that competency in basic and advanced life support generally declines 6 months after completing training (36). Previous research underscores the importance of continuing training and education in BLS and ALS to enhance the effectiveness of CPR (17, 37).

In Ecuador, knowledge deficiencies in life support have been observed in other groups of health workers, such as doctors (38), highlighting a structural problem where key stakeholders, including universities, health institutions, and ultimately the health system, do not assign the necessary importance to life support preparation. We believe that addressing the deficiency of knowledge in life support among nursing personnel in Ecuador can be initiated by making life support a mandatory requirement in all higher education institutions. This should follow a gradual approach, beginning in the first year of the university degree and continuing throughout subsequent years of training based on international guidelines. Additionally, practical courses should be included annually to maintain optimal competence over the four and a half years of training (11). Furthermore, health institutions and health authorities must concentrate efforts and allocate resources to ensure the periodic training of nursing professionals. This will enable them to update their knowledge and achieve optimal results.

### 4.1 Limitations

This study presents several limitations that could affect the robustness of its conclusions. First, the self-report design of the study inherently introduces a risk of selection bias, as nursing professionals with a particular interest in life support and emergency care may have been more inclined to participate. Furthermore, the assessment system employed to gauge participants’ knowledge was specifically developed by the investigators for this study, potentially affecting the generalizability of the results and any comparisons with findings from other research. However, rigorous steps were taken to ensure the validity of the instrument, including an expert review to standardize the weighting of questions and the use of a decimal-type numerical rating scale that evaluated each question individually.

Another limitation lies in the questionnaire, which solely measures the theoretical knowledge of basic and advanced life support. This presents an avenue for future research that could incorporate assessments of practical skills among Ecuadorian nurses. In addition, we were unable to provide an accurate picture of the possible influence of formal “official certification” training in basic and advanced life support on the level of knowledge because the exact period from the time participants obtained their certificate to the time of data collection was not considered.

To prevent duplicate responses, the survey utilized the features of the Google Forms platform to allow only one response per device, which may have unintentionally restricted multiple legitimate respondents who share a device. However, we could not control the behavior of the participants during the completion of the surveys, so there was a possibility that participants may have copied within the knowledge test.

Lastly, social desirability bias could have influenced the results, particularly if respondents were concerned about receiving low knowledge scores. Although we emphasized the anonymity of the data and the importance of honest responses, this may not have entirely mitigated the impact of this bias.

## 5 Conclusion

Ecuadorian nursing professionals demonstrate alarmingly insufficient general levels of theoretical knowledge in both basic and advanced life support. Although knowledge of BLS was marginally better than that of advanced life support, participants did not achieve an acceptable average score in either case.

This research reinforces previous postulates regarding the positive impact and importance of continuous training and education in life support contexts, as evidenced by higher scores among these groups.

In the Latin American context, the development and inclusion of nursing professionals in basic and advanced life support training programs are of paramount importance.

## Data availability statement

The datasets presented in this study can be found in online repositories. The names of the repository/repositories and accession number(s) can be found at DOI: 10.6084/m9.figshare.23506539.

## Ethics statement

All collected information was anonymized, and participation was voluntary. Ethical approval was obtained from the Ethics Committee of the Hospital San Francisco de Quito, under the code CEISH151 HGSF-2023-011, ensuring compliance with ethical guidelines and standards.

## Author contributions

JI-C: Conceptualization, Data curation, Formal analysis, Funding acquisition, Investigation, Methodology, Project administration, Resources, Software, Supervision, Visualization, Writing – original draft, Writing – review & editing. FA: Conceptualization, Investigation, Resources, Validation, Writing – original draft. ED-S: Writing – original draft. NA: Investigation, Resources, Validation, Writing – original draft. MR: Investigation, Resources, Validation, Writing – original draft. PN-L: Data curation, Investigation, Resources, Validation, Visualization, Writing – original draft. AM: Data curation, Investigation, Methodology, Resources, Validation, Writing – original draft. JJ-S: Data curation, Investigation, Methodology, Resources, Validation, Writing – original draft. DC: Investigation, Resources, Validation, Visualization, Writing – original draft. BG: Investigation, Resources, Validation, Visualization, Writing – original draft. NG: Data curation, Investigation, Resources, Validation, Writing – original draft.
