# Supplementary material for: Assessment of preparedness and proficiency in basic and advanced life support among nursing professionals: a cross-sectional study
Source: Front Med (Lausanne). 2024 Jan 22;11:1328573. doi: 10.3389/fmed.2024.1328573 (PMC10840996; doi:10.3389/fmed.2024.1328573)
Supplement: Supplementary file 1 [file Data_Sheet_1.docx]

Supplementary Material

Assessment of preparedness and proficiency in basic and advanced life support among nursing professionals: A cross-sectional study

Juan S. Izquierdo-Condoy^1*^, Fabián D. Arias Rodríguez^2^, Erick Duque-Sánchez^1^, Nicolas Alegria M.^1^, Marlon Rojas Cadena^1^, Patricio Naranjo-Lara^1^, Alexander Puglla Mendoza^1^, Jackson Jima-Sanmartín^1^, Dayana Andrade Casanova^1^, Balbina García^1^, Natalia Castaño Giraldo^3^

*** Correspondence:** Juan S. Izquierdo-Condoy: juan1izquierdo11@gmail.com

# Questionary

**“Knowledge in basic and advanced life support in Ecuadorian nurses”**

**Section 1:**

- - - 1. Gender: Male ( ) Female ( ) I'd rather not say it ( )
      2. Age (please provide in years): ___ years.
      3. What is your academic level?

University degree ( ) Specialist ( ) Martes degree ( ) PhD ( )

- - - 1. Where do you perform your duties?

Health Center ( ) Basic Hospital ( ) General Hospital ( ) Specialty Hospital ( )

- - - 1. To which sector does your workplace belong?

Public Sector ( ) Private Sector ( )

- - - 1. In which area do you work?

Outpatient ( ) Emergency ( ) Intensive Care ( ) Anesthesiology ( ) Surgery ( ) Cardiology ( )
Internal Medicine ( ) Orthopedics ( ) Other ( ) Specify: ____

7. How long have you been working?

Less than 3 years ( ) Between 3 to 6 years ( ) Between 6 to 10 years ( ) More than 10 years ( )

**Section 2:**

1. Did you receive formal training during your college education on life support (BLS or ALS)?

Yes ( ) No ( )

1. Have you been certified through a BLS course?

Yes ( ) No ( )

1. Have you had experience performing BLS resuscitation?

Less than 3 times ( ) Between 3 and 6 times ( ) More than 6 times ( )

1. Do you feel you have sufficient knowledge of BLS?

Yes ( ) No ( )

1. Have you been certified through an ALS course?

Yes ( ) No ( )

1. Have you had experience of performing resuscitation with ALS?

Less than 3 times ( ) Between 3 and 6 times ( ) More than 6 times ( )

1. Do you feel you have sufficient knowledge of ALS?

Yes ( ) No ( )

1. Have you done any extracurricular activities to train in life support?

Scientific Articles ( ) Official Guidelines ( ) Evidence-based Abstracts ( ) Videos ( )
Other ( ) None ( )

**Section 3 - BLS Knowledge:**

What is the correct sequence of actions during the application of BLS on an adult victim with a rescuer?

1. Evaluate the person, call the emergency department, take care of yourself, CPR, defibrillate.
2. Protect yourself, evaluate the person, call the emergency department, defibrillate, perform CPR.
3. Protect yourself, evaluate the person, perform CPR, call the emergency department, defibrillate.
4. Protect yourself, evaluate the person, call the emergency department, perform CPR, defibrillate.
5. How can you check the victim's responsiveness?
6. Shake the person, tap his or her shoulder hard, and talk out loudly.
7. Shake the person and feel arterial pulses.
8. Shake the person, listen to their breathing, talk out loudly.
9. Shake the person, touch your leg hard, talk out loudly.
10. What is the main action to keep the airways open?
11. Head tilt/chin descent maneuver
12. Head tilt/chin lift maneuver
13. Head rectification/chin descent manoeuvre
14. Head rectification/chin lift manoeuvre
15. What is the compression-to-ventilation ratio in an adult victim with one rescuer?
16. 30/2
17. 30/1
18. 40/2
19. 30/4
20. What is the correct hand placement for chest compressions in adult resuscitation?
21. Use the heel of one hand on the upper half of the breastbone in the center of the chest and place the other hand on top of the first.
22. Use the heel of one hand on the lower half of the breastbone in the center of the chest and place the other hand on top of the first.
23. Use the heel of one hand on the lower half of the breastbone in the center of the chest and place the other hand beside the first.
24. Use the heel of one hand on the upper half of the breastbone on the side of the chest and place the other hand on top of the first.
25. How deep should the chest be compressed during chest compressions in adult resuscitation?
26. 1-2 cm
27. 3-4 cm
28. 5-6 cm
29. 7-8 cm
30. Which of the following is NOT a complication of performing BLS?
31. Rib or sternal fractures
32. Liver lesions
33. Pneumothorax
34. Pneumomediastinum
35. What is the maximum time allowed to evaluate pulse and breathing in an unconscious person?
36. 20 seconds
37. 30 seconds
38. 10 seconds
39. 15 seconds
40. At what chest compression fraction does the probability of return of spontaneous circulation increase?
41. 40%
42. 50%
43. 60%
44. 70%
45. In a person who has regained a pulse after CPR but continues to be unresponsive or is not breathing, how many rescue ventilations should be provided per minute?
46. 18 – 20 rpm
47. 5 – 6 rpm
48. 10 – 12 rpm
49. Do not ventilate

**Section 4 - ALS Knowledge:**

1. What is the recommended dose of epinephrine for cardiac arrest?
2. 1.0 mg (1:10000) IV/IO
3. 0.1 mg (1:100000) IV/IO
4. 10.0 mg (1:1000) IV/IO
5. 1 ampoule (1:1000) in 10 ml of saline.
6. Which of the following is a shockable rhythm?
7. Ventricular fibrillation
8. Ventricular tachycardia (no pulse)
9. Torsades de Pointes
10. All of the above
11. Which of the following antiarrhythmic medications is NOT recommended for persistent ventricular fibrillation or pulseless ventricular tachycardia?
12. Amiodarone
13. Lidocaine
14. Atropine
15. Epinephrine
16. What is the maximum dose of atropine for symptomatic bradycardia?
17. 2 mg
18. 3 mg
19. 4 mg
20. 5 mg
21. What is the maximum dose of amiodarone?
22. 300 mg
23. 400 mg
24. 450 mg
25. 500 mg
26. Which of the following are causes of pulseless electrical activity?
27. Hypovolemia
28. Hypoglycemia
29. Hypothermia
30. Acidosis
31. Hypoxia
32. Which information is crucial to obtain for all individuals presenting with a stroke?
33. Date of birth
34. Glycosylated hemoglobin
35. Bilateral arm blood pressure
36. Last known well time
37. In post-cardiac arrest care, which of the following respiratory parameters is NOT considered stable?
38. Maintain 16 rpm
39. Oximetry 92-98%
40. PaCO2 35-45 mmHg
41. Waveform capnography
42. Induced hypothermia is known to aid in brain recovery after cardiac arrest. What is the target core body temperature range during induced hypothermia?
43. 30 – 34 °C
44. 32 – 36 °C
45. 33 – 36 °C
46. 32 – 35 °C
47. What is the initial shock energy dose during defibrillation?
48. 160-240 J
49. 100-150 J
50. 120-200 J
51. 200-300 J
